# Supplementary material for: Identification and Functional Characterisation of Two Oat UDP-Glucosyltransferases Involved in Deoxynivalenol Detoxification
Source: Toxins (Basel). 2022 Jun 30;14(7):446. doi: 10.3390/toxins14070446 (PMC9318758; doi:10.3390/toxins14070446)
Supplement: Supplementary file 1 [file toxins-14-00446-s001.zip › Supplemetary File S3_chromatograms.pdf]

Supplementary File S3: Chromatograms of substrate and product of whole E.coli cells treated with DON

Qualitative Plot Window Report

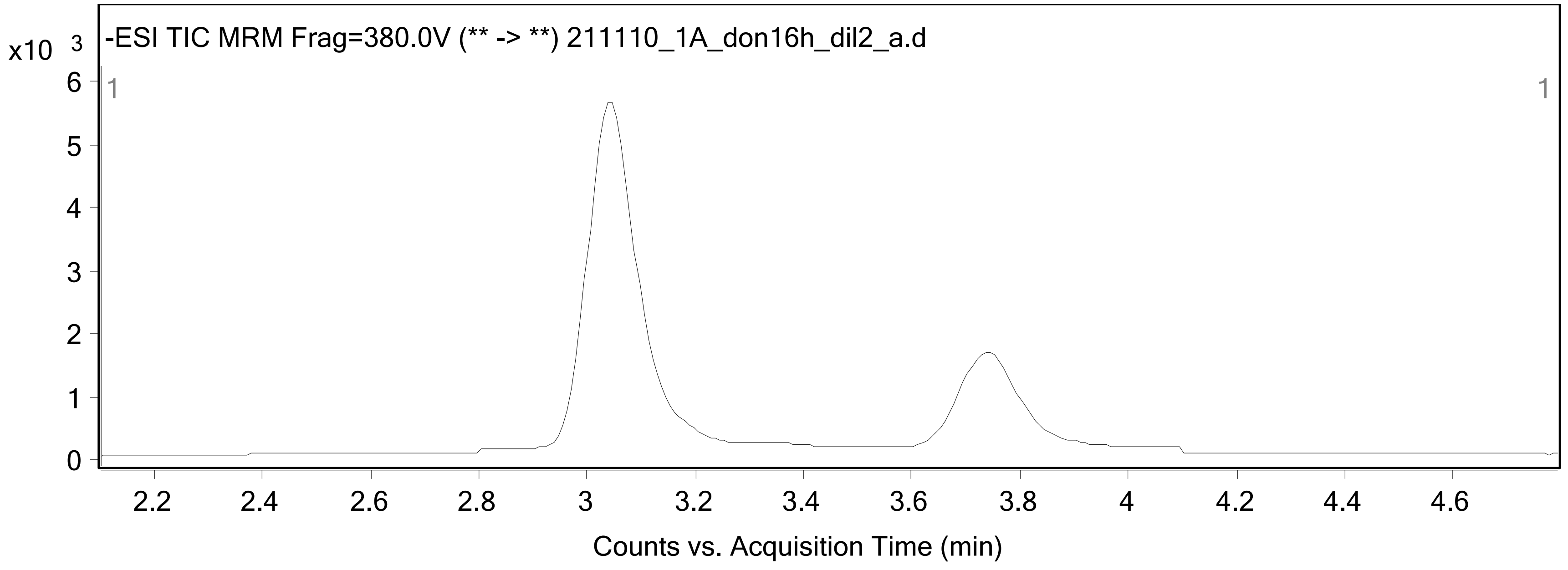

Figure 1: E.coli expressing AsUGT1 (transformant 1) after 16h treatment with DON

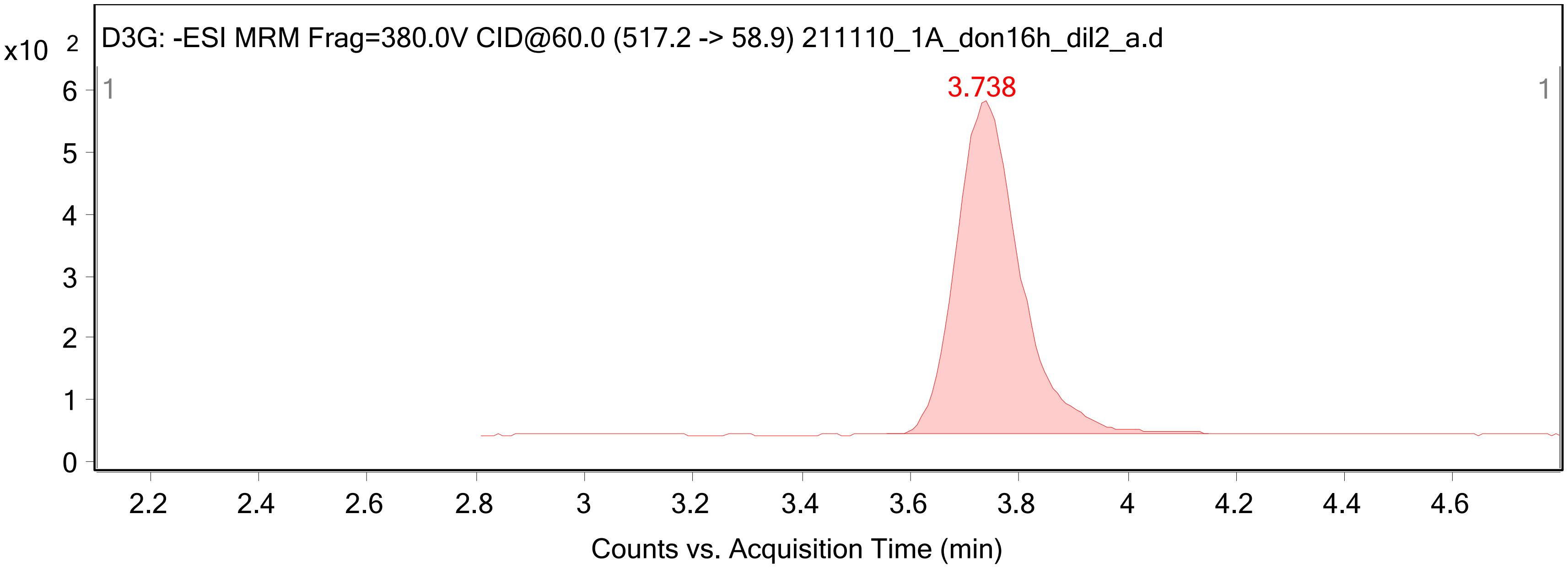

Figure 2: E.coli expressing AsUGT1 (transformant 1) after 16h treatment with DON. (D3G, CID@60)

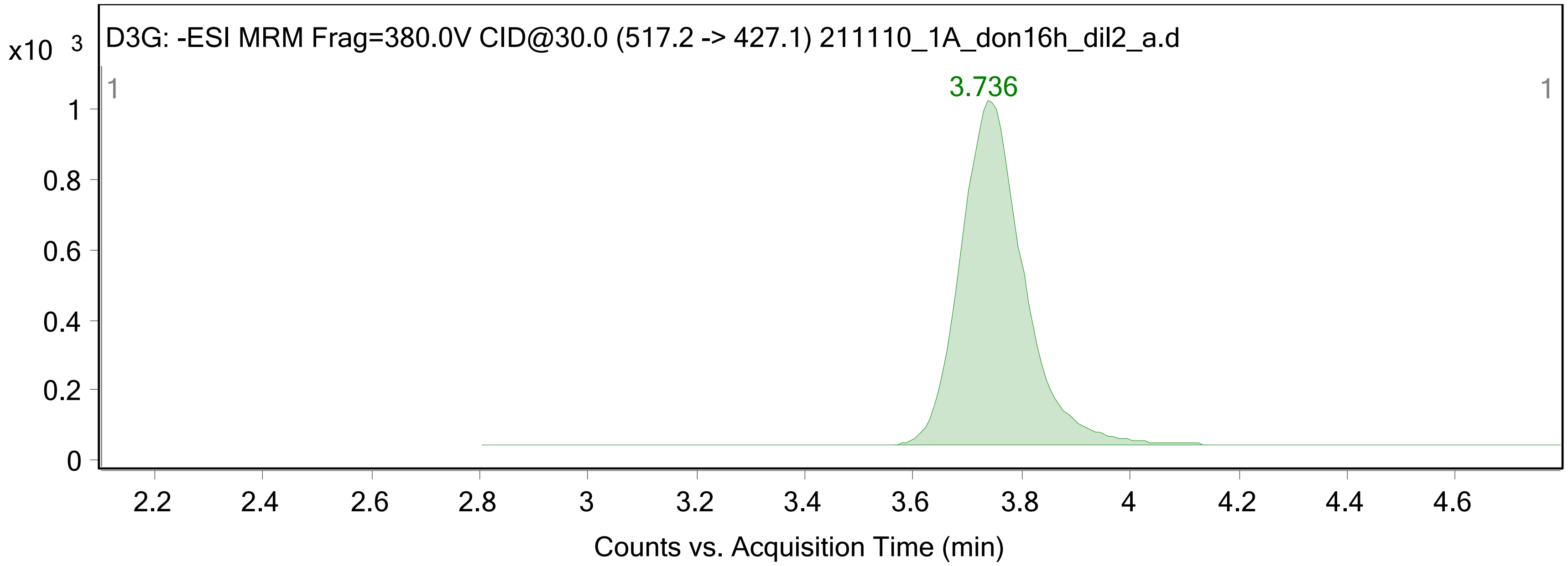

Figure 3: E.coli expressing AsUGT1 (transformant 1) after 16h treatment with DON. (D3G, CID@30)

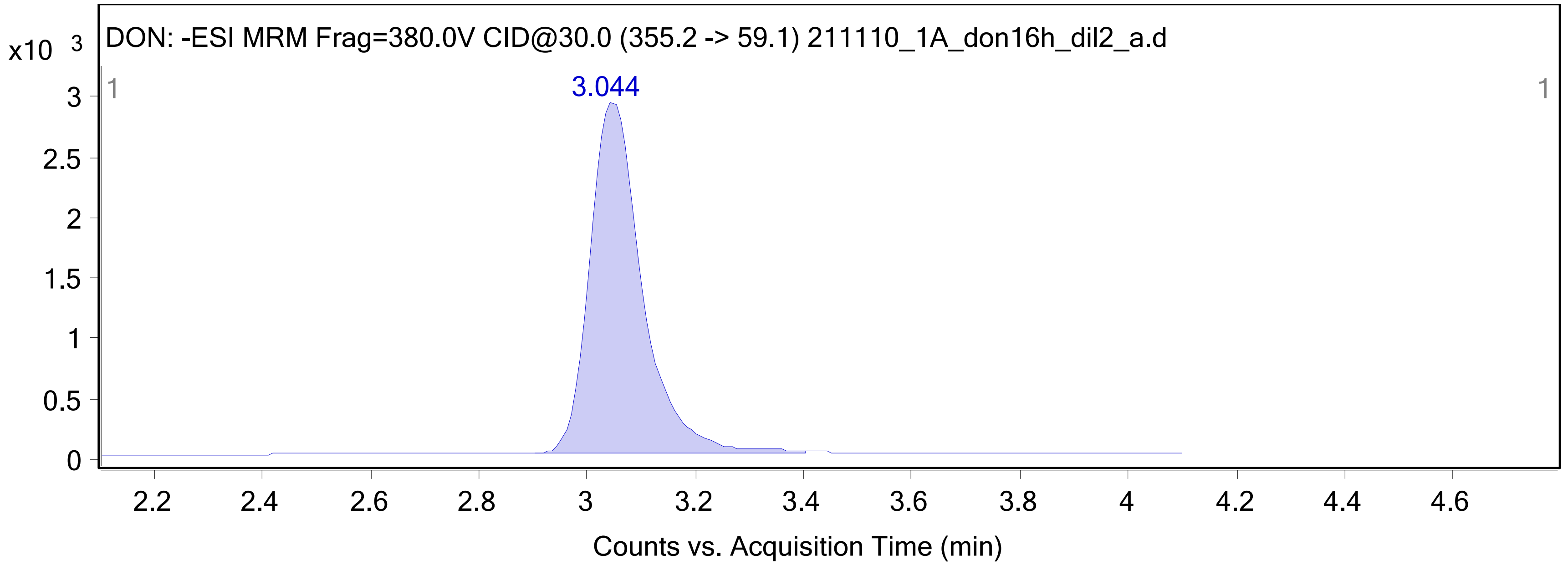

Figure 4: E.coli expressing AsUGT1 (transformant 1) after 16h treatment with DON. (DON, CID@30)

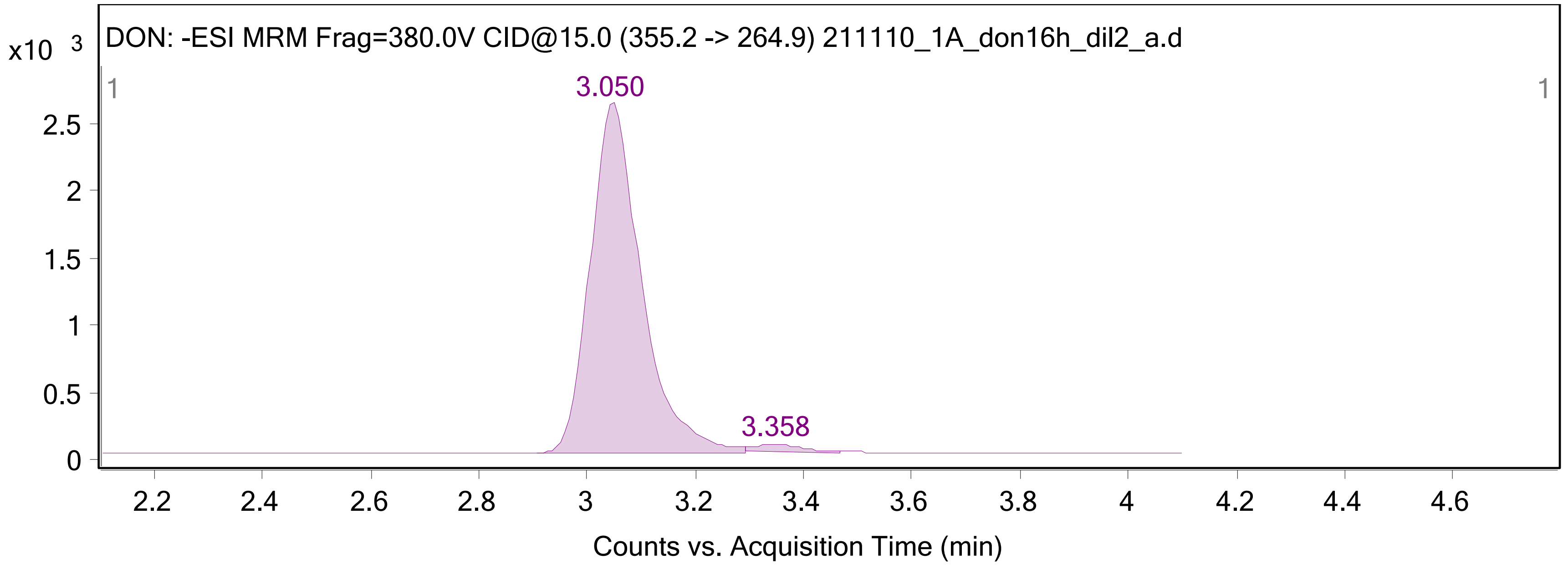

Figure 5: E.coli expressing AsUGT1 (transformant 1) after 16h treatment with DON. (DON, CID@15)

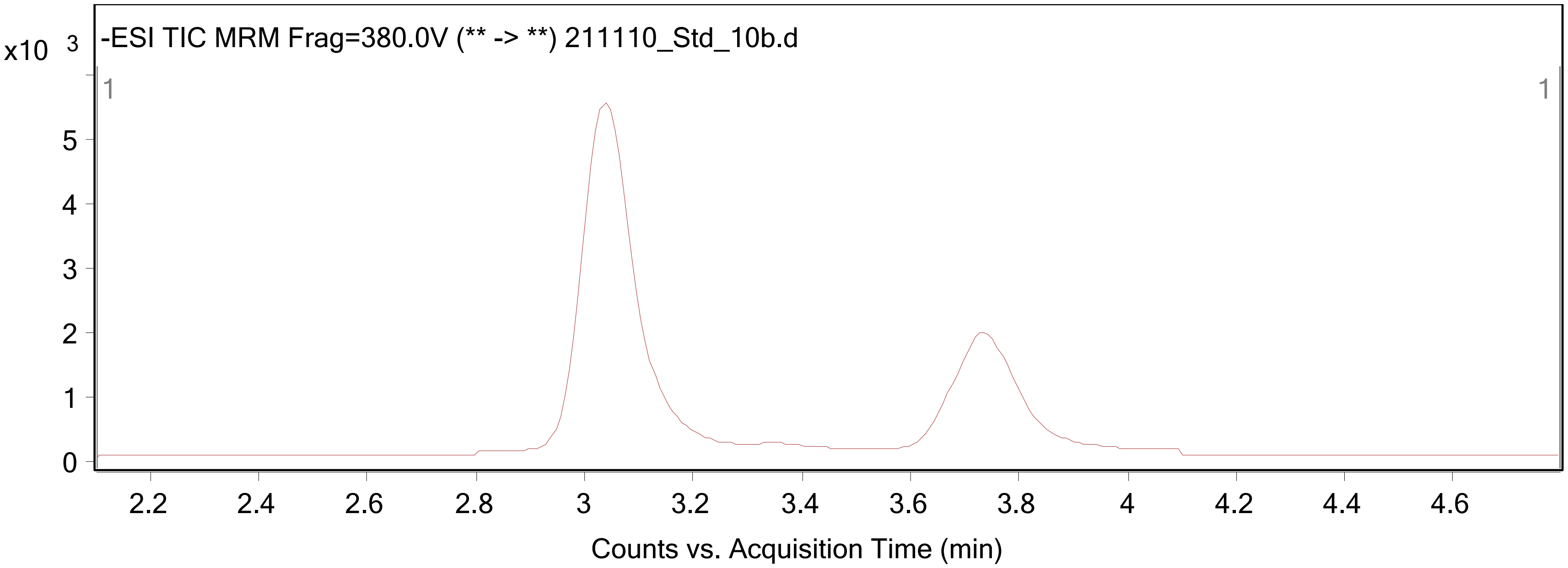

Figure 6: Standard 10mkg/ml DON/ 10 mkg/ml D3G

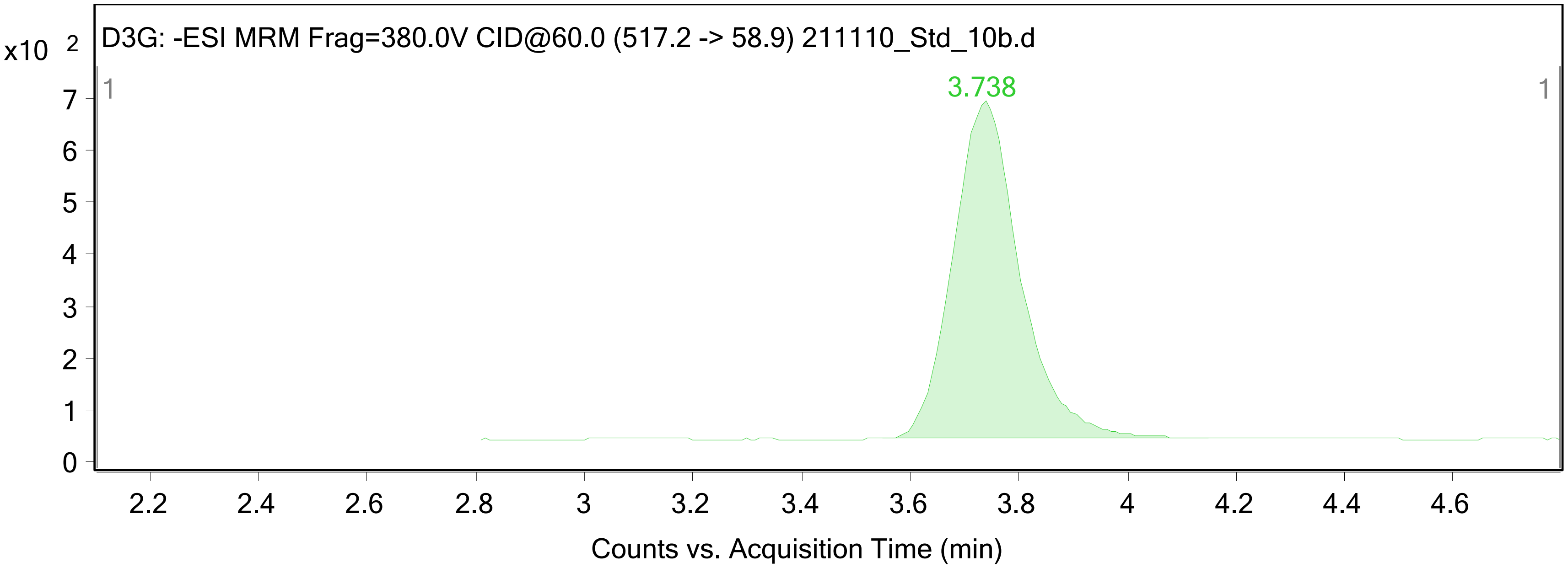

Figure 7: Standard 10mkg/ml DON/ 10 mkg/ml D3G (D3G, CID@60)

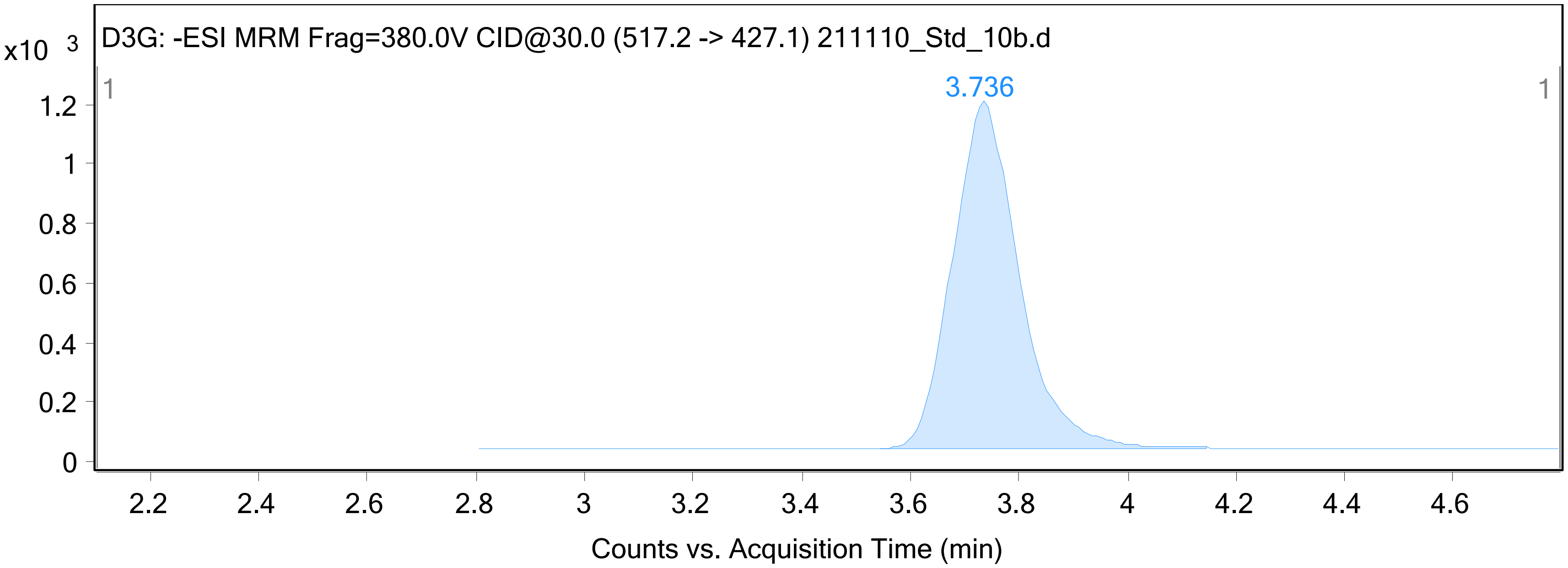

Figure 8: Standard 10mkg/ml DON/ 10 mkg/ml D3G (D3G, CID@30)

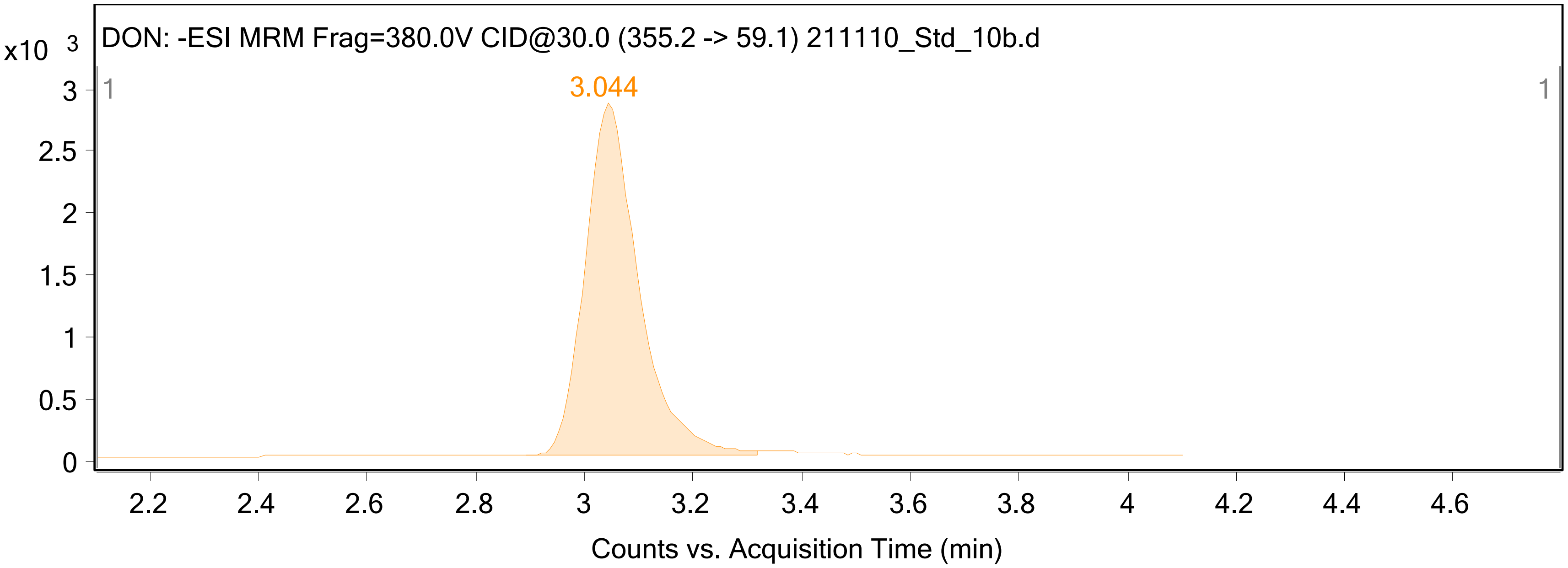

Figure 9: Standard 10mkg/ml DON/ 10 mkg/ml D3G (DON, CID@30)

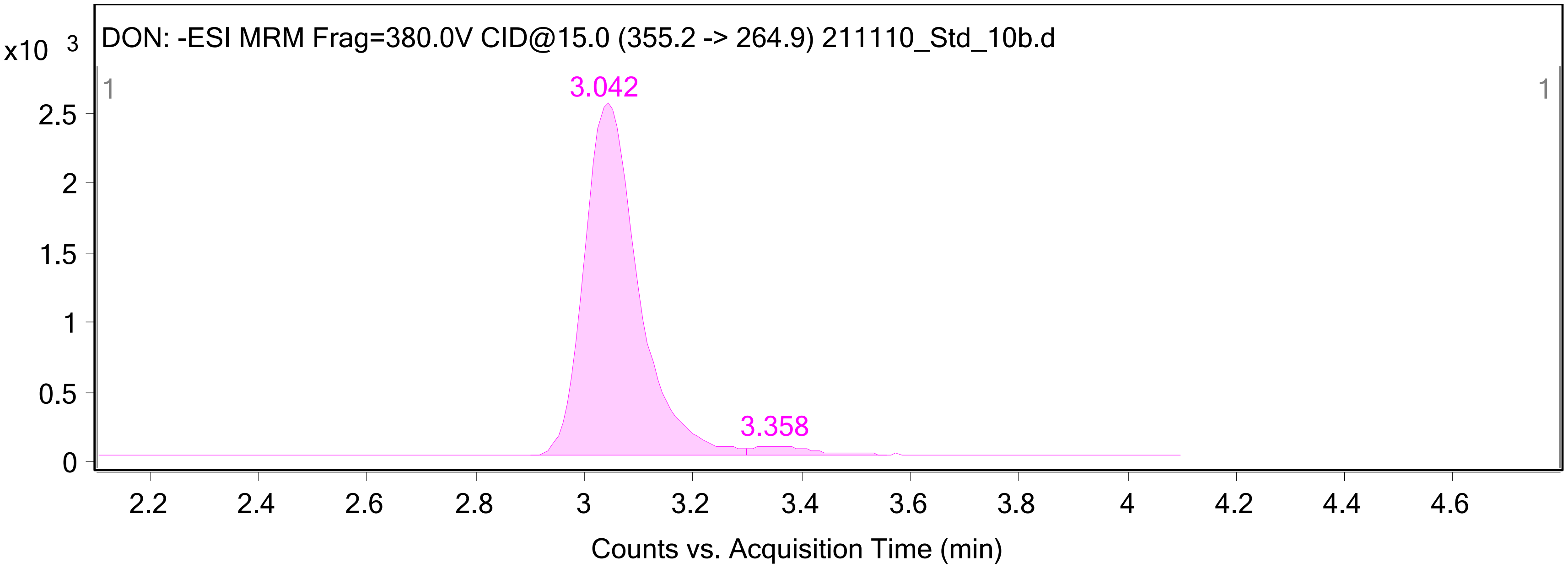

Figure 10: Standard 10mkg/ml DON/ 10 mkg/ml D3G (DON, CID@15)
