# Supplementary material for: Identification and Functional Characterisation of Two Oat UDP-Glucosyltransferases Involved in Deoxynivalenol Detoxification
Source: Toxins (Basel). 2022 Jun 30;14(7):446. doi: 10.3390/toxins14070446 (PMC9318758; doi:10.3390/toxins14070446)
Supplement: Supplementary file 1 [file toxins-14-00446-s001.zip › toxins-1779186-Supplementary.pdf]

Supplementary Materials: Identification and Functional Characterisation of Two Oat UDP-Glucosyltransferases Involved in Deoxynivalenol Detoxification

Alfia Khairullina, Nikos Tsardakas Renhuldt, Gerlinde Wiesenberger, Johan Bentzer, David B. Collinge, Gerhard Adam and Leif Bülow

AsUGT1

GCCC**AAGCTT**ATGGAGACTACTTCTACTGTAACTGGAGCCGTGACTGTTACCACGTCAGC
CTCTGTTAGTCATGGAGGAGGCGGCGACGGTGGAGCCTCTGTTTTCTTCCTTCCGTTCCC
TGGTGCGCAGGGCCATACGAATCCTATGCTACAGTTCGGCCGTCGTCTGGCGTACCACGG
CCTACGTCCAACGTTGGTCGTTACCCGTTATGTCCTTTCTACGGCCGATGCACCACCAGG
AGATCCATTTCGTGTAGCGGCGATAAGTGACGGTTTTGATGCTGGCGGCATGGCTTCCTG
CCCCGATTACGCTGATTACGTCAGTCGTATGGAGGCCGTCGGTTCAGAAACTCTGCGTGA
ACTACTACTGAGTGAAGCTCGTGCGGGACGTCCCGTTAGTGTCCTGGTGTATGATCCACA
CCTACCATGGGCCTTACCTGTAGCTCGTGAAGCAGGCGTGGCTACTGCTGCCTTTTTCAG
CCAACCTTGTGCTGTCGATGTGATCTATGGAGAAGTTTGGGCCGGCCGTATGGCTCTACC
GGCCACAGATGGGCGTGAGTTAGTCGCACGTGGAGCGCTATCAGTTGAACTTGGCCCAGA
GGATATGCCCCCTTTTGTCGCTGTCCCCGAGTCTCAACCGGTGTTAACTAAAACCTCCAT
ACGTCAGTTCGAGGGTTTGGAGGAAGCAGACGACGTCTTGGTAAATAGTTTCCGTGACTT
TGAGCCGAAGGAAGCCAAGTATATGGAGTTGACCTGGCGTGCCAAGATGGTTGGGCCTAC
TCTTCCCTCTTTCTATCTTGATGATGATCGTTTACCGTCTAATAAGTCATACGGATTTAA
TTTGTTTTCCGGTGATGCCCCTTGTATGGACTGGCTGGAAAAACAGTCAATCTCTTCTGT
GGTACTTGTCTCATACGGGACGGTGTCTAATTATGACACGTCTCAGCTTGAAGAGCTGGG
CAATGGTCTATGCAACTCAGGCAAGCCCTTTATATGGGTTGTTCGTTCCAACGAAGCACA
TAAGCTAACTGAAGAACTGAAAATGAAGTGCGAAAAGATTGGCCTTATAGTCTCCTGGTG
TCCACAATTGGAGGTGCTTGCCCACAAGGCAATCGGGTGCTTCGTTACCCACTGTGGCTG
GAATTCAACTCTTGAAGCGGTCGCCTCCGGGGTTCCACTGGTGGGTATTCCTGATTGGGC
CGATCAGCCCACTATCTCAAAATACGTCGAAAGTGTTTGGGGAATGGGGGTCGTCCGTAA
AGGAGAAAATGGCTTTATCGGTAGCGGCGACATTGAGAGTTGCATTCGTGAGGTGATGGA
CGGCGAGCGTAAAGATGAGTATAAGCGTAATGCGATGAAGTGGATGCAGAAAGCTAAGGA
AGCAATGCGTGAGGGGGGTAGTTCTGATATACATATAATAGAGTTTGCAGCTAAGTATAA
ATAA**GCGGCCGC**TAAACTAT

AsUGT2

GCCC**AAGCTT**ATGGAAACGACCAGTACCGTTACACGTGCAGTAACAGTGACGACATCCGC
GTCTGTCTCACACGGTGGCGGAGACGGGGGAGCAAACGTCTTTTTTCTGCCATTCCCAGG
AGCGCAGGGTCATACCAACCCAATGCTTCAATTTGGCCGTCGTCTAGCCTATCACGGACT
TCGTCCCACCTTAGTGGTAACTCGTTATGTCCTTTCTACTGCTGATGCTCCACCTGGAGA
TCCCTTCCGTGTGGCAGCCTTTTCTGACGGCTTCGACGCAGGCGGTATGGCTAGTTGTCC
AGATTACGCCGATTATCTGTCCCGTATGGAAACAGTTGGTAGCGAGACCCTACGTGAATT
GTTATTGTCAGAGGCGCGTGCGGGCCGTCCGGTGCGTGTGTTAGTTTACGACCCGCACTT
ACCTTGGGCCCTACCTGTCGCACGTGAGGCAGGGGTCGCGACAGCCGCTTTCTTTTCCCA
ACCATGCGCCGTAGATATCATTTACGGGGAACTTTGGGCTGGCCGTATGGCCCTGCCAGC
AACCGATGGGCGTGAACTAGTAGCCCGTGGGGCTCTTTCCGTCGAACTAGGGCCAGATGA
TATGCCCCCTTTTGTAGCAGTCCCAGAGTGGCAGCCGGTGTTGACAAAGACGTGTATACG
TCAATTCGAAGGTTTAGATGAAGCCGACGATGTTTTAGTAAACTCTTTTCGTGACTTCGA
GCCTAAGGAAGCGAAATATATGGAATTGAGGTGGCGTGCCAAGATGATAGGCCCTACTCT
TCCTAGTTTTTATCTTGATGATGATCGTTTACCCTCAAATAAGTCATACGGATTTAACCT
GTTTAGCGGCGATGCGCCTTGCATGGACTGGCTAGATAAGCAATCCATCTCTAGCGTTGT
GTTGGTGTCTTACGGGACTGTCTCTAATTATGATACAAGTCAACTAGAAGAATTAGGAAA

TGGCTTGTGTAACAGCGGTAAGCCATTTATCTGGGTAGTGCGTAGCAATGAGGCGCATAA
GCTGACCGAGGAATTAAAGATGAAATGCGAAAAGATCGGCCTTATTGTTAGTTGGTGCCC
CCAATTAGAGGTCTTGGCCCACAAGGCTATTGGGTGTTTTGTAACCCATTGTGGGTGGAA
CTCAACATTAGAAGCTGTAGCGTCAGGGGTACCCTTGGTTGGCATACCCGACTGGGCAGA
CCAACCAACTATCTCTAAATACGTTGAATCTGTTTGGGATATGGGTGTGCGTGTAAGGAA
ACGTGAAAACGGCTTTATCTGGTCACGTGACATTAAATCCTGCATACGTGAAGTAATGAA
CGGCGAGCGTAAGGACGAGTACAAACGTAATGCAATGAAATGGATGCAAAAAGCCAAAGA
AGCAATGCGTGATGGGGGCTCCTCCGATATTCACATAGTAGAGTTCGCGGCAAAATACAA
GTAA**GCGGCCGC**TAAACTAT

Figure S1. Sequences of oat UGT genes, codon-optimized for expression in *S. cerevisiae* (Flanking HindIII and NotI restriction enzyme sites are marked with bold script, Stop and Start codon marked with colors).

Table S1. Primers and probes used for the analysis of UGT expression in oat.

Gene	Primer/probe name	Sequence
Tubulin-alfa	Tub-a forward	GAG GTG GAA CTG GCT CTG
	Tub-a reverse	GAC ACT GTT GTA TGG CTC AAC
	Tub-a probe	/5HEX/TCA CCT CAG /ZEN/GTC TCC ACC TCT GTT /3IABkFQ/
AsUGT1	AsUGT1 forward	GTG GAT GGA ACT CAA CAT TGG AG
	AsUGT1 reverse	GGT CAG CCC AAT CTG GAA TAC
	AsUGT1 probe	/56-FAM/CCG TTG CTA /ZEN/GCG GTG TAC CTC TTG /3IABkFQ/
AsUGT2	AsUGT2 forward	GAC CAA CCC ACC ATC TCA AA
	AsUGT2 reverse	CCC ATT CAT CAC CTC TCT AAT ACA A
	AsUGT probe	/56-FAM/TTT CTC TCT /ZEN/TCC GCA CTC GCA CAC /3IABkFQ/
